# NK cell activation and CD4^+^ T cell α4β7 expression are associated with susceptibility to HIV-1

**DOI:** 10.1172/JCI187992

**Published:** 2025-05-08

**Authors:** Kawthar Machmach, Kombo F. N’guessan, Rohit Farmer, Sucheta Godbole, Dohoon Kim, Lauren McCormick, Noemia S. Lima, Amy R. Henry, Farida Laboune, Isabella Swafford, Sydney K. Mika, Bonnie M. Slike, Jeffrey R. Currier, Leigh Anne Eller, Julie A. Ake, Sandhya Vasan, Merlin L. Robb, Shelly J. Krebs, Daniel C. Douek, Dominic Paquin-Proulx, for the RV217 Study Group

**Affiliations:** 1 US Military HIV Research Program, Center for Infectious Disease Research, Walter Reed Army Institute of Research, Silver Spring, Maryland, USA.; 2 Henry M. Jackson Foundation for the Advancement of Military Medicine Inc, Bethesda, Maryland, USA.; 3 Human Immunology Section, Vaccine Research Center, National Institute of Allergy and Infectious Diseases (NIAID), NIH, Bethesda, Maryland, USA.; 4 Viral Diseases Program, Center for Infectious Disease Research, Walter Reed Army Institute of Research, Silver Spring, Maryland, USA.

**Keywords:** AIDS/HIV, Infectious disease, NK cells, NKT cells, T cells

## Abstract

We leveraged specimens from the RV217 prospective study that enrolled participants at high risk of HIV-1 acquisition to investigate how NK cells, conventional T cells, and unconventional T cells influence HIV-1 acquisition. We observed low levels of α4β7 expression on memory CD4^+^ T cells and invariant NK T (iNKT) cells, 2 cell types highly susceptible to HIV-1 infection, in highly exposed seronegative (HESN) compared with highly exposed seroconverter (HESC) participants. NK cells from HESN individuals had higher levels of α4β7 than did those from HESC individuals, presented a quiescent phenotype, and had a higher capacity to respond to opsonized target cells. We also measured translocated microbial products in plasma and found differences in phylum distribution between HESN and HESC participants that were associated with the immune phenotypes affecting the risk of HIV-1 acquisition. Finally, a logistic regression model combining features of NK cell activation, α4β7 expression on memory CD4^+^ T cells, and T-box expressed in T cells (Tbet) expression by iNKT cells achieved the highest accuracy in identifying HESN and HESC participants. This immune signature, consisting of increased α4β7 on cells susceptible to HIV infection combined with higher NK cell activation and lower gut-homing potential, could affect the efficacy of HIV-1 prevention strategies such as vaccines.

## Abstract 2

We propose a model whereby increased α4β7 expression on cells susceptible to HIV infection combined with higher NK cell activation and lower gut-homing potential is associated with an increased risk of HIV acquisition.

## Introduction

According to the World Health Organization, there were an estimated 39 million people with HIV (PWH) at the end of 2022, and an estimated 1.3 million people acquired HIV during that year (1). The factors influencing the risk of acquiring HIV-1 are multiple and include exposure to other sexually transmitted infection (2), behavior (3), genetic determinants such as CCR5-D32 homozygous mutation (4, 5), and immune activation (6, 7). HIV-exposed seronegative (HESN) individuals are a unique model to study determinants of HIV-1 susceptibility. Several studies have compared the immune landscape in HESN and highly exposed seroconverter (HESC) individuals. CD4^+^ T cells are the primary target of HIV, and thus comparing their phenotype between HESC and HESN has been of great interest. Lower levels of the activation markers CD38 and HLA-DR as well as the proliferation marker Ki67 have been reported in HESN individuals (7). Similarly, lower levels of CD38 and HLA-DR on CD8^+^ T cells have also been reported in HESN participants of the iPrEx trial (6). HESN individuals were also reported to have increased levels of regulatory CD4^+^ T cells (Tregs) compared with HESC individuals (8). α4β7 is an integrin that binds to mucosal vascular addressin cell adhesion molecule 1 (MAdCAM-1) and is involved in trafficking of lymphocytes to the gastrointestinal mucosa (9). CD4^+^ T cells expressing α4β7 are particularly susceptible to HIV infection and are targeted early during acute infection (10–12). Higher expression of α4β7 has been associated with an increased risk of HIV-1 acquisition in a cohort of high-risk women from KwaZulu-Natal in South Africa (13). However, this result has not been replicated in cohorts of men who have sex with men (MSM) or people who inject drugs (PWID) (14).

NK cells are an important component of innate immunity due to their role in virus and tumor surveillance. Several lines of evidence show that NK cells can affect HIV disease progression. Killer Ig-like receptors (KIRs) and HLA combination can either delay (15) or promote disease progression (16). KIR^+^ NK cells have also been suggested to mediate immune pressure selecting for HIV sequence polymorphisms (17). NK cell activation has also been associated with HIV post-treatment virological control (18). Furthermore, diversity of surface receptor expression on NK cells was reported to affect the susceptibility to HIV acquisition (19). NK cells from HESN participants have also been reported to have higher levels of constitutive degranulation, as measured by surface CD107a levels, compared with controls (20). Another study reported higher levels of the activation markers CD69 and HLA-DR on NK cells in HESC compared with HESN individuals (21).

Unconventional T cells do not recognize peptides presented by MHC and include CD1d-restricted invariant NK T (iNKT) cells, MR1-restricted mucosa-associated invariant T (MAIT) cells, as well as γδ T cells (22). These cells are profoundly affected by chronic HIV infection (23–27). Furthermore, anti-HIV activity has been reported for MAIT cells (28) and γδ T cells (29). iNKT cells also have the capacity to recognize HIV-infected DCs, but this is normally inhibited by Nef- and Vpu-mediated downregulation of CD1d (30). Both CD4^+^ iNKT cells and γδ T cells have been shown to be susceptible to HIV infection in vivo (31, 32). How unconventional T cells might affect HIV acquisition has not been studied, to our knowledge. Furthermore, the interactions between various cell types might be important for determining HIV susceptibility, and this has not been thoroughly investigated.

RV217 was a prospective natural history study involving individuals at high risk for HIV acquisition in East Africa and Thailand (33). Here, we used cryopreserved PBMCs from 25 RV217 participants from Thailand who acquired HIV-1 from a pre-seroconversion time point matched with 74 Thai participants who did not acquire HIV-1 for the duration of the study to investigate by flow cytometry how NK cells, conventional T cells, and unconventional T cells influence HIV-1 acquisition. We also determined the composition of translocated microbial products in plasma in a subset of participants and identified differences in phylum distribution that were in turn associated with the immune phenotype affecting the risk of HIV-1 acquisition. Finally, we applied logistic regression model analysis to identify the minimal combination of markers that could identify HESC and HESN individuals. Together, our results suggest that translocated microbial products might influence the phenotype of HIV-1–susceptible cells as well as NK cells that in turn affect HIV-1 acquisition. This immune signature could affect the efficacy of HIV prevention strategies such as vaccines.

## Results

RV217 is a prospective natural history study involving over 3,000 individuals at high risk for HIV acquisition in 4 countries (33). A total of 155 participants acquired HIV-1 during the course of the study. Thus, RV217 offers a unique opportunity to investigate the determinants of HIV acquisition. Cryopreserved PBMCs from Thai RV217 study participants were used to investigate T cell and NK cell phenotypes in 25 HESC individuals prior to seroconversion and 74 HESN individuals for comparative analysis (Table 1). The participants included were predominantly young (median age of 23 years) for both groups, male or transgender.

### Increased frequencies of α4β7-expressing memory CD4^+^ T cells and iNKT cells in HESC individuals prior to HIV acquisition.

**Figure 1:**
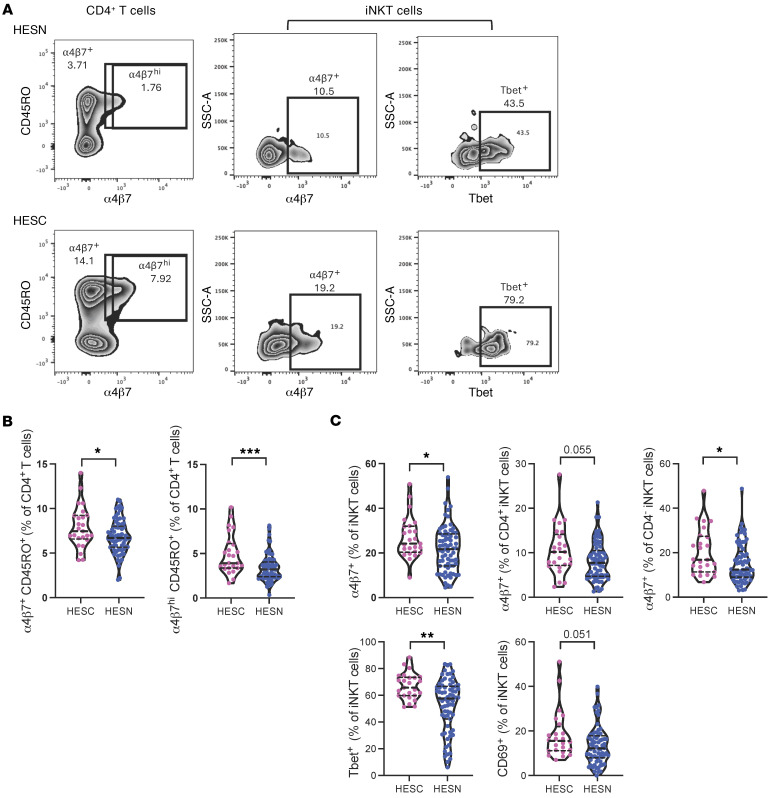
Conventional and unconventional T cell phenotypes in HESN and HESC individuals. (**A**) Representative flow cytometry plots. (**B**) Levels of α4β7^+^ and α4β7^hi^CD45RO^+^CD4^+^ T cells. (**C**) Levels of α4β7^+^ iNKT cells, α4β7^+^CD4^+^ iNKT cells, α4β7^+^CD4^–^ iNKT cells, Tbet^+^ iNKT cells, and CD69^+^ iNKT cells in HESN and HESC individuals prior to HIV-1 acquisition. *n* = 25 HESC individuals; *n* = 74 HESN individuals. *P < 0.05, **P < 0.01, and ***P < 0.001, by Mann-Whitney *U* test. Horizontal lines represent the median with IQR.

First, we determined the frequency and phenotype of total and memory subsets of CD4^+^ and CD8^+^ T cells by flow cytometry in HESC and HESN individuals prior to HIV acquisition (Supplemental Figure 1A; supplemental material available online with this article; https://doi.org/10.1172/JCI187992DS1). We found no difference in CD4^+^ or CD8^+^ T cell frequencies between HESC and HESN individuals prior to HIV acquisition (Supplemental Figure 1B). Similarly, we observed no difference between HESC and HESN participants in the levels of activation markers among total CD4^+^ T cells and CD8^+^ T cells (Supplemental Figure 2). However, we observed increased frequencies of α4β7-expressing CD4^+^ memory T cells in HESC participants (α4β7^+^, *P* = 0.049; α4β7^hi^, *P* < 0.001) (Figure 1, A and B).

We subsequently investigated whether unconventional T cell frequencies and phenotypes differed between HESC and HESN individuals prior to acquisition. We did not observe differences in unconventional T cell frequencies between HESC and HESN individuals (Supplemental Figure 1B). Similar to memory CD4^+^ T cells, our results showed that α4β7-expressing iNKT cells were increased in HESC compared with HESN participants (*P* = 0.03) (Figure 1, A and C). This difference was observed in both the CD4^+^ and CD4^–^ subsets of iNKT cells. We also observed an increase in T-box expressed in T cells (Tbet)-expressing iNKT cells in HESC individuals (*P* = 0.002) (Figure 1, A and C) and a trend for higher levels of CD69 expression by iNKT cells in HESC individuals (*P* = 0.051) (Figure 1C). We observed no difference in MAIT or γδT cell phenotypes between HESC and HESN individuals (data not shown). Our data identify an increase in frequencies of α4β7-expressing memory CD4^+^ T cells and iNKT cells in HESC participants prior to HIV acquisition compared with HESN participants, and suggest a contribution of these cell types in susceptibility to HIV acquisition.

### Activated and dysfunctional NK cell phenotype in HESC individuals.

**Figure 2:**
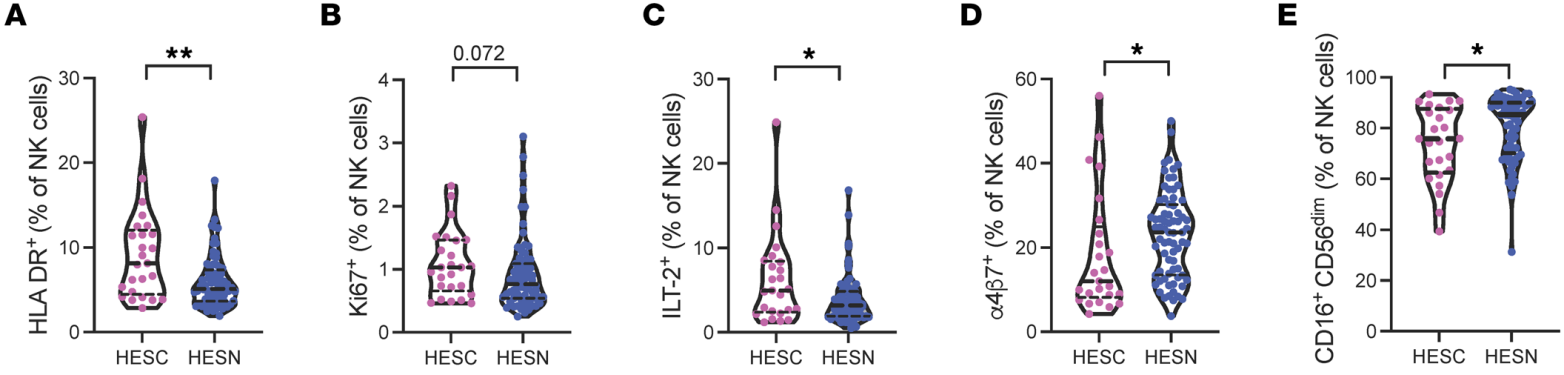
NK cell phenotype in HESN and HESC individuals. Violin plots showing expression levels of HLA-DR (**A**), Ki67 (**B**), ILT-2 (**C**), α4β7 (**D**), and CD16 (**E**) in NK cells from HESC individuals (*n* = 25) prior to HIV-1 acquisition and in HESN individuals (*n* = 74). *P < 0.05 and **P < 0.01, by Mann-Whitney *U* test. Horizontal lines represent the median with IQR.

Next, to determine whether the NK cell phenotype and/or distribution are altered in HESC compared with HESN individuals, we used a 26 multiparameter flow cytometry panel to deeply characterize these cells (34) (Supplemental Figure 3A). First, we analyzed the distribution of the 3 canonical NK cell subsets based on the expression of CD56. The distribution of the 3 NK cell subsets, immature CD56^bright^, cytotoxic and mature CD56^dim^, and CD56^lo^ NK cells was similar in both groups (Supplemental Figure 3B). NK cells from HESC individuals showed a trend toward displaying a more mature phenotype, indicated by higher expression of the maturation marker NKG2C (*P* = 0.06), and reduced expression of the receptor NKG2A (*P* = 0.09) (Supplemental Figure 3C). NK cells from HESC individuals also showed significantly higher levels of the activation marker HLA-DR (*P* = 0.003) and a trend toward higher proliferation, as measured by Ki67 expression (*P* = 0.07) (Supplemental Figure 4 and Figure 2, A and B). We analyzed the expression of the inhibitory receptor Ig-like transcript 2 (ILT-2), a receptor recently shown to be expressed by dysfunctional NK cells during chronic hepatitis B (35). In our study, we observed that the frequency of ILT-2^+^ NK cells was significantly higher in HESC participants than in HESN participants (*P* = 0.035) (Supplemental Figure 4 and Figure 2C). However, we observed no difference in programmed cell death 1 (PD-1) expression by NK cells between HESC and HESN participants (data not shown, *P* = 0.63).

The ability of NK cells to migrate and participate in the early mucosal antiviral responses is crucial. Therefore, we analyzed the expression levels of the gut-homing marker α4β7 and observed significantly decreased expression of α4β7 by NK cells (*P* = 0.011) in HESC compared with HESN individuals (Supplemental Figure 4 and Figure 2D). NK cells from HESC individuals also showed decreased expression of the NK cell–activating receptor CD16 (*P* = 0.043), whose engagement triggers NK cell cytotoxicity and the production of cytokines and chemokines (Supplemental Figure 4 and Figure 2E).

**Figure 3:**
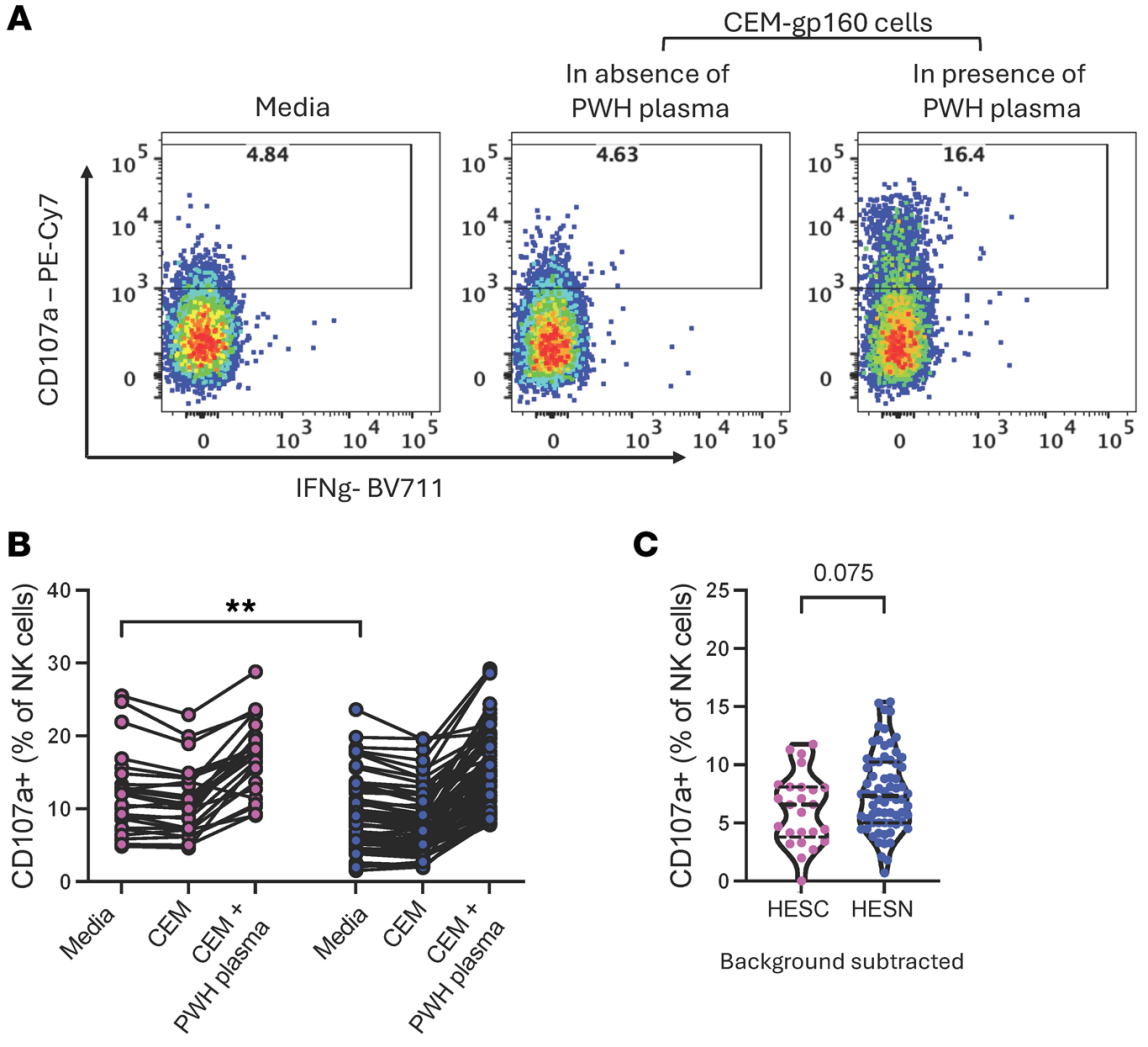
NK cell degranulation ability in HESN and HESC individuals. (**A**) Representative flow cytometry plots. (**B**) Levels of the degranulation marker CD107a in unstimulated or stimulated NK cells in coculture with gp160-expressing cells previously incubated in the presence or absence of PWH plasma. (**C**) Levels of induction of the degranulation marker CD107a after subtracting the value of CEM-gp160 without PWH plasma. Horizontal lines represent the median with IQR. *n* = 25 HESC individuals prior to HIV-1 acquisition; *n* = 74 HESN individuals. **P < 0.01, by Mann-Whitney *U* test.

These results suggested a difference in NK cell function between HESN and HESC individuals. Therefore, we tested the capacity of NK cells to respond to gp160-expressing cells opsonized with plasma from an individual with HIV-1. Interestingly, NK cells from HESC participants had a trend for lower induction of the degranulation marker CD107a in response to opsonized gp160-expressing cells (*P* = 0.075) (Figure 3, A–C), despite showing higher spontaneous levels in the absence of target cells (*P* = 0.003) (Figure 3, A and B). Our data suggest that prior to seroconversion, NK cells from HESC participants might have a refractory phenotype impairing the cells from migrating to mucosal tissue and limiting their contribution to an effective innate antiviral response at the site of HIV exposure.

### Differential composition of translocated microbial products between HESN and HESC individuals is associated with a change in immune cell phenotype.

The microbiome is known to interact with the immune system and shape its composition (36, 37). To determine whether the microbiome might contribute to the differences in immune phenotypes we observed between HESN and HESC participants, we performed shotgun deep sequencing of cell-free DNA and RNA fragments in plasma in a subset of participants (38). We observed no difference in the proportion of translocated microbial products from archaea, bacteria, eukaryota, or viruses in the DNA fraction (Supplemental Figure 5A). However, HESC individuals had a higher proportion of archaea as well as a trend toward a higher proportion of bacteria compared with HESN individuals when we analyzed the RNA fraction (Supplemental Figure 5B). Next, we conducted a subanalysis using a gut microbial reference data set of 3,594 species (39) and found higher gut-associated bacterial RNA in HESC compared with HESN participants (Supplemental Figure 5, C and D). To gain initial insight as to whether differences exist in the composition of translocated microbial products, we calculated the Shannon diversity, richness, and evenness scores, grouping the organisms into their respective kingdoms. We found no differences between HESC and HESN participants (data not shown). We then conducted principal component analysis (PCA), again grouping the organisms into their respective kingdoms. No separation was observed for any kingdom using either DNA or RNA (Supplemental Figure 6, A and B). Next, we tested whether the generated PCAs were associated with the immune phenotypes identified by flow cytometry (Supplemental Figure 6, C and D). Several PCAs generated from viral DNA were associated with features of NK, iNKT, and memory CD4^+^ T cells. PCAs from archaea DNA were associated with α4β7 expression by NK, iNKT, and memory CD4^+^ T cells, whereas PCAs of bacterial DNA and RNA were associated with features of NK and iNKT cells.

**Figure 4:**
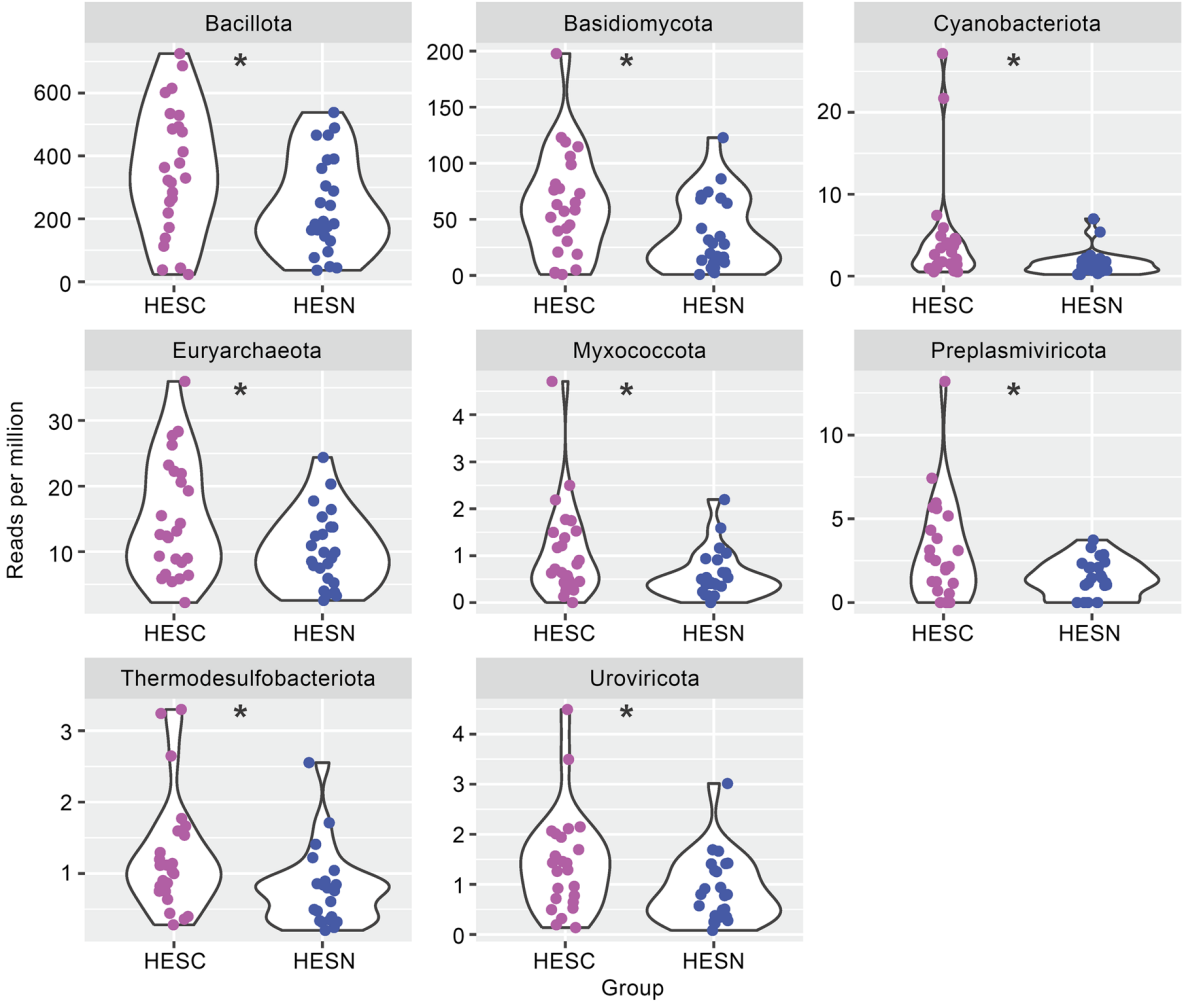
Composition of the translocated microbial products in HESN and HESC individuals prior to seroconversion. Violin plots showing the relative abundance as RPM of selected phyla that were significantly different between HESC individuals prior to HIV-1 acquisition (*n* = 25) and HESN individuals (*n* = 24). *P < 0.05, by 2-sided *t* test.

**Figure 5:**
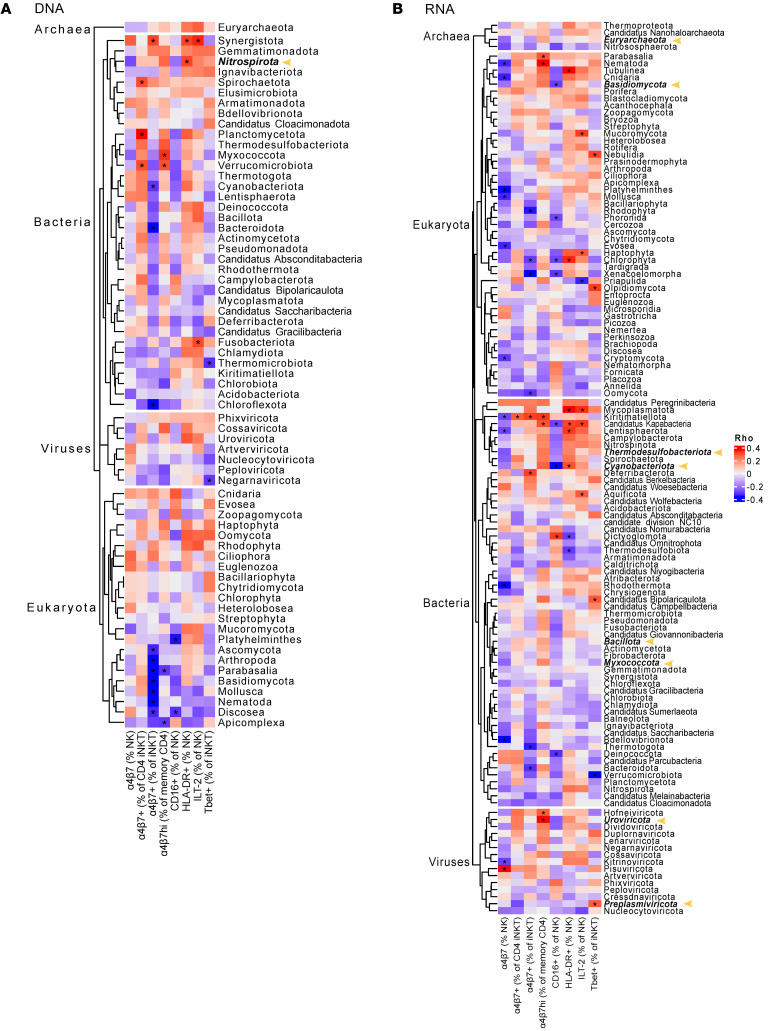
Associations between translocated microbial products and immune phenotype. Heatmap showing the Spearman rho values for the association between translocated microbial products at the phylum level and immune phenotype for DNA (**A**) and RNA (**B**). *P* values below 0.05 (Spearman’s correlation test) are indicated by an asterisk. Phyla with a difference in relative abundance between HESC and HESN individuals are indicated by a yellow arrowhead. *n* = 49.

To further characterize the difference in translocated microbial products, we compared the relative proportion of translocated microbial RNA at the phylum level. A total of 8 phyla were significantly enriched in HESC compared with HESN individuals, including Basidiomycota, cyanobacteria, and *Preplasmiviricota* (Figure 4 and Supporting Data Values file). Finally, we tested for associations between translocated microbial products and the expression levels of the markers found to be differentially expressed between HESC and HESN individuals. Analysis of the translocated DNA fraction showed that the proportion of several eukaryotes was inversely associated with α4β7 expression by iNKT cells (Figure 5A). The proportion of translocated RNA of Basidiomycota and cyanobacteria was inversely associated with CD16 expression by NK cells (Figure 5B). Additionally, the proportion of translocated cyanobacteria RNA also associated with HLA-DR expression by NK cells. Tbet expression by iNKT cells was associated with the *Preplasmiviricota* RNA proportion. Interestingly, the RNA proportion of *Candidatus kapabacteria* trended higher in HESC participants (Supporting Data Values file) and was associated with ILT-2^+^ NK cells, HLA-DR^+^ NK cells, and α4β7^hi^-expressing memory CD4^+^ T cells and was inversely associated with CD16 expression by NK cells. Overall, this suggests that differences in the distribution of translocated microbial products might contribute to the immune phenotype associated with greater susceptibility to HIV acquisition.

### The levels of HIV-1 target cell and NK cell activation are associated with HIV-1 acquisition.

Next, we explored the relationship between the immune phenotypes that were different between HESN and HESC participants in our univariate analysis and early viral replication during acute infection and disease progression. None of the identified parameters was significantly associated with peak or set-point viral load as well as the CD4^+^ to CD8^+^ T cell ratio at set-point viral load or after 1–2 years after HIV acquisition (data not shown).

**Figure 6:**
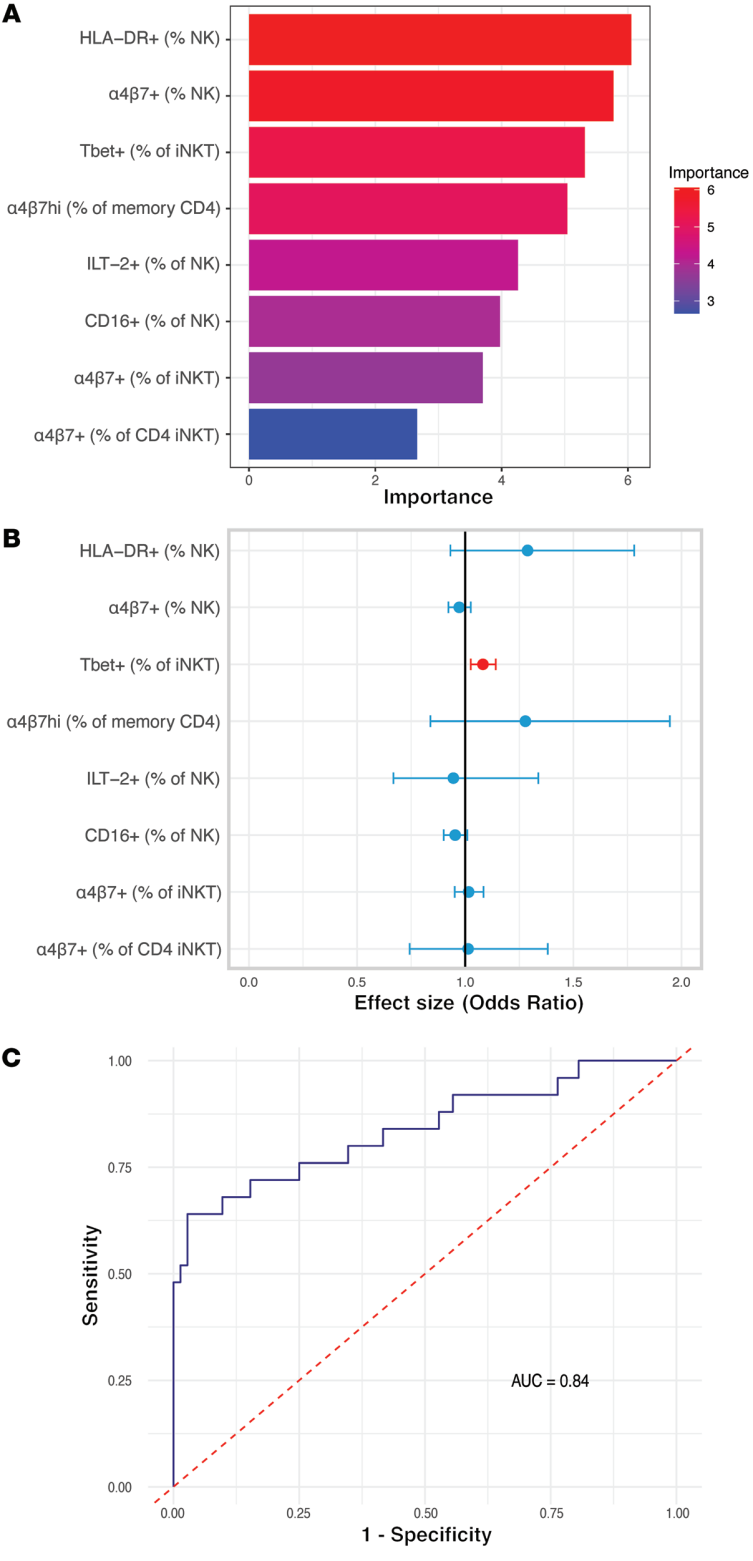
Statistical model estimating the ability of the immune phenotype to predict seroconversion. (**A**) Logistic regression analysis estimating the statistical significance and level to which each marker might increase or decrease the odds of acquiring HIV. (**B**) Random forest analysis indicating the importance of each marker in predicting HESC and HESN status on the basis of the mean decrease in the Gini coefficient. The marker with a significant effect is indicated in red. Lines represent the 95% CI. (**C**) ROC curve of the optimized logistic regression model using 4 predictors: HLA-DR^+^ (percentage of NK cells), Tbet^+^ (percentage of iNKT cells), α4β7hi (percentage of CD45RO CD4^+^ T cells), and CD16^+^ (percentage of NK cells).

To investigate the relationship between immune cell phenotypes prior to HIV acquisition as predictors for seroconversion, we performed random forest and logistic regression analyses using the immune phenotypes that were different between HESN and HESC participants in our univariate analysis. The random forest analysis indicates the importance of each immune cell phenotype in predicting HESC and HESN status. Our data show that, among the phenotypes identified as significantly different between HESC and HESN individuals, the percentages of HLA-DR^+^ NK cells, α4β7^+^ NK cells, and Tbet^+^ iNKT cells were the most important predictors of HESC and HESN status (Figure 6A). Logistic regression analysis revealed that each percentage increase in Tbet^+^ iNKT cells increased the odds of HIV acquisition by 8% (Figure 6B). No other marker was found to significantly affect the odds of HIV acquisition independently. Finally, to consider interactions between the immune phenotypes, we generated an optimized regression model. This model used only the percentages of HLA-DR^+^ NK cells, Tbet^+^ iNKT cells, α4β7hi CD4^+^ memory T cells, and CD16^+^ NK cells and had a sensitivity of 64%, a specificity of 95.83%, and an accuracy of 87.63% in predicting whether study participants were HESNs or HESCs with an area under the receiving operator curve (AUC) of 0.84 (Figure 6C). Thus, a combination of markers indicative of HIV-1 target cells and NK cell activation provided the best classification of study participants, suggesting that both target cell availability and NK cell innate immune response were influencing HIV-1 acquisition.

## Discussion

In this study, we compared the phenotypes of circulating NK and T cells between highly exposed Thai RV217 study participants who acquired HIV-1 or did not acquire it. The prospective RV217 cohort included a sampling of study participants that allowed us to assess the phenotype of immune cells before HIV-1 acquisition. Furthermore, we selected HESN participants to match the demographics of the HESC participants to minimize potential confounders.

Relative to HESN individuals, we found that participants who acquired HIV-1 had higher levels of α4β7 on memory CD4^+^ T cells and iNKT cells. This suggests that memory CD4^+^ T cells and iNKT cells from HESC individuals might have a greater capacity to migrate to mucosal tissues by binding to MadCam-1 (9) and position these cells at the sites where HIV-1 transmission can occur. In support of this, the frequency of peripheral α4β7^hi^ CD4^+^ T cell has been reported to correlate with the frequency of these cells in the female reproductive tract (13). This could be particularly important, as α4β7 memory CD4^+^ T cells and CD4^+^ iNKT cells have been reported to be preferential targets for HIV replication during acute infection (12, 31). Thus, a greater availability of cells highly susceptible to infection at the point of viral entry might facilitate early replication of HIV. Our results are similar to those of another study that was conducted in highly exposed females in South Africa (13) and to those of SIV studies (40, 41), highlighting the importance of α4β7 in HIV acquisition. The importance of α4β7-expressing memory CD4^+^ T cell in HIV acquisition was further confirmed, as this was 1 of the 4 features selected by our optimized model. One possible explanation is the observation that the V2 loop of the gp120 HIV envelope induces activation signals that promote HIV infection following binding to α4β7 (42). Another mechanism proposed to explain the role of α4β7 in HIV acquisition is that α4β7-expressing CD4^+^ T cells have high levels of the HIV coreceptor CCR5 (11). However, our results are in contrast with the findings from another study (14). One possible explanation is that circulating viruses might differ in their capacity to bind to α4β7. In Thailand, CRF01_AE is predominant, and a CRF01_AE primary virus showed high binding to α4β7 in vitro (43). Similarly, Sivro et al. showed that the effect of α4β7 on HIV acquisition was higher for viruses containing a motif associated with V2 binding (13). A recent study found mixed associations between α4β7 expression on CD4^+^ T cells and HIV acquisition in various cohorts (44), suggesting a complex interplay between several factors. Our approach allowed us to identified other immune features that affected HIV acquisition, together with α4β7 expression on memory CD4^+^ T cells. Tbet expression by iNKT cell was also found to be a key factor associated with HIV acquisition. It is possible that Tbet^+^ iNKT cells are also highly susceptible to HIV infection. However, further work is needed to understand the link between iNKT cells expressing Tbet and HIV acquisition.

In contrast to memory CD4^+^ T cells and iNKT cells, we observed lower expression levels of α4β7 on NK cells from HESC participants. The lower capacity of NK cells to migrate to the gut mucosa might prevent them from having an effective early innate immune response that would contain viral replication locally before systemic spread. Contrary to other studies (6, 7), we did not observe differences in CD4^+^ or CD8^+^ T cell activation. However, we found increased NK cell activation in HESC individuals, as evidenced by higher levels of surface HLA-DR and spontaneous degranulation. Higher levels of NK cell activation in HESC women have also been previously reported (21). NK cells from HESC individuals also had higher levels of the inhibitory receptor ILT-2 and lower levels of CD16. Disintegrin and metalloprotease-17 (ADAM17) is known to cleave cell-surface CD16 following NK cell activation (45). These results further support the observation that NK cells from HESC individuals are more activated than those from HESN individuals. This higher baseline activation and expression of the inhibitory receptor might make NK cells from HESC individuals refractory to further stimulation. Our optimized model selected 2 features related to NK cell activation (HLA-DR and CD16 expression), further confirming the importance of NK cell activation in susceptibility to HIV acquisition.

Microbiome dysbiosis has been shown to influence SIV acquisition (46). Thus, we postulated that differences in microbiome composition between HESC and HESN individuals could explain in part the differences in immune cell phenotypes we observed. One limitation of our study is that no tissue or stool samples were collected for microbiome analysis. Instead, we measured translocated microbial products found in plasma. The translocated microbial products have been shown to influence CD4^+^ T cell reconstitution following antiretroviral therapy (ART) initiation (38). In our study, HESC participants had higher plasma levels of RNA coming from archaea and gut-associated bacteria. Furthermore, 8 phyla were also more represented in HESC participants, and some of these correlated with the immune phenotypes that we found to be significantly different between HESN and HESC participants. Thus, we speculate that the microbiome might influence HIV acquisition by modulating key immune cell populations. However, more work is needed to determine whether the microbiome directly influences the immune phenotype associated with HIV acquisition.

In conclusion, we propose a model in which increased α4β7 expression on cells susceptible to HIV infection, combined with higher NK cell activation, inhibitory receptor expression, and lower gut-homing potential, is associated with an increased risk of HIV acquisition. Our work also suggests that the composition of the microbiome influences the immune phenotypes associated with HIV-1 acquisition. The effect of HIV prevention strategies, such as vaccines, on this immune signature should be considered, as it may affect their efficacy.

## Methods

### Sex as a biological variable.

Both males and females as well as transgender individuals were included in this study. The sex distribution is representative of the state of the HIV-1 epidemic in Thailand at the time of RV217 study completion.

### Study participants.

The RV217 study has been described previously (33). Briefly, the RV217 Study enrolled consenting adults from key populations at 4 clinical research sites in Kenya, Uganda, Tanzania, and Thailand. To be included in the RV217 ECHO study, participants had to meet one of the following criteria within the previous 3 months: had exchanged goods for sex, had unprotected sex with a known HIV^+^ partner, had unprotected sex with 3 or more partners, or had symptoms of a sexually transmitted infection. Study participants were screened twice weekly for HIV-1 infection through finger pricks analyzed by a nucleic acid amplification test (NAAT) (Aptima HIV-1 RNA Qualitative test, Hologic Inc.). The cases presented in this study are a selected set from a group of 25 RV217 participants from Thailand for whom cryopreserved PBMCs were available prior to HIV acquisition matched for age, sex, or gender, and risk behavior, with 3 individuals also from Thailand who did not acquire HIV during the course of the study (Table 1).

### Viral RNA quantification in plasma.

HIV-1 RNA levels were measured using the RealTime HIV-1 Assay (m2000 RealTime System, Abbott Molecular).

### Flow cytometry.

Cryopreserved PBMCs were thawed in RPMI medium containing benzonase nuclease and 20% FBS. Following the washes, cells were stained with LIVE/DEAD Fixable Aqua Dead Cell Stain Kit (Thermo Fisher Scientific, catalog L34957) in PBS for 30 minutes at room temperature. FcR blocking was conducted for 15 minutes at room temperature with 10% normal mouse IgG (Thermo Fisher Scientific, catalog OB2040-09) in staining buffer (PBS containing 0.1% NaN_3_ and 1.7% BSA). Cell-surface staining was conducted using cocktails of fluorescently labeled antibodies (from BD Biosciences unless otherwise indicated). For T cell analysis, MR1 tetramer (the MR1 tetramer technology was developed jointly by James McCluskey, Jamie Rossjohn, and David Fairli [ref. 47], and the material was produced by the NIH Tetramer Core Facility as permitted to be distributed by the University of Melbourne); Va24 BB515 (Beckman Coulter, catalog IM1589); CCR6 BB630P (custom); CD69 BB660-P2 (custom); CXCR5 RB780 (catalog 755631); α4β7 AF647 (clone Act-1, NIH AIDS Reagent Program, Division of AIDS, NIAID, NIH; catalog 11718) from A.A. Ansari, labeled using the Alexa Fluor 647 antibody labeling kit from Invitrogen (Thermo Fisher Scientific); CD161 R718 (catalog 751652); CD8 APC Cy 7 (catalog 557760); HLA-DR BV480 (catalog 566113); CD19 BV570 (custom); CD14 BV570 (custom); CCR7 BV650 (catalog 566756); X-pan TCRgd BV711 (catalog 745505); CD45RO BV786 (catalog 564290); CD57 BUV395 (catalog 567621); CD16 BUV496 (catalog 612944); X-Vd2 TCR BUV563 (catalog 748582); CD56 BUV615-P (catalog 751349); PD-1 BUV661 (catalog 750260); CD38 BUV737 (catalog 612824); CD4 BUV805 (catalog 612887); Vb11 PE (Beckman, catalog IM2290); Vα7.2 PE-Dazzle594 (BioLegend, catalog 351730); CD3 PE Cy5.5 (Thermo Fisher Scientific, catalog 35-0036-42); and TCRd1 PE Cy7 (Thermo Fisher Scientific, catalog 25-5679-42), in staining buffer with Brilliant Stain Buffer (Thermo Fisher Scientific, catalog 00-4409-42) were incubated for 30 minutes at room temperature. Intracellular staining was performed after fixation and permeabilization using the FOXP3/Transcription Factor Staining Buffer Set (Invitrogen, Thermo Fisher Scientific, catalog 00-5523-00) and a cocktail of the following fluorescent antibodies against intracellular proteins: granzyme B BV604 (custom), T-bet PE Cy5 (custom), and Ki67 BV750 (custom). For NK cell analysis, cells were stained as previously described (34). Flow cytometric data were collected on a FACSymphony A5 flow cytometer (BD) and analyzed using FlowJo, version 10.8.1 software for Mac OS (BD). A complete list of antibodies used can be found in Supplemental Table 1.

### NK cell in vitro functional assay.

The clade B HXB2 gp160 fragment (2568bp; GenBank NP_057856.1) was created by in vitro synthesis with codon optimization of the gp120 region (bases 1–1530) and native sequence for bases 1531–2568 retaining the Rev response element of gp41. A Kozak sequence was appended to the 5′ end, and the entire construct was subcloned as a NotI/ApaI fragment into pcDNA3.1(+)/Hygro. This plasmid was linearized and transfected by electroporation into CEM.NK^R^ cells. After antibiotic selection, sorting and cloning a cell line expressing uniform levels of clade B HXB2 gp160 was obtained. CEM.NK^R^ cells were obtained from the NIAID AIDS Reagent Repository (ARP-458). ARP-458 is a variant of the CEM cell line that is resistant to NK cell–mediated lysis and expresses CD4 and CXCR4 (48).

To analyze NK cell capacity to mediate ADCC, CEM.NKR target cells were preincubated with and without plasma from an individual with HIV for 30 minutes at 37°C before adding PBMCs from the study participants at an effector-to-target ratio of 1:5 in the presence of anti-CD107a antibody (H4A3, BD Biosciences). Cytokine production and changes in CD107a expression in stimulated (with plasma) relative to unstimulated (without plasma) total NK cells served as a surrogate measure of cytotoxicity toward antibody-coated target cells. To evaluate spontaneous ex vivo NK cell activity, PBMCs were left untreated for 6 hours in the presence of anti-CD107a antibody. Brefeldin A (Thermo Fisher Scientific, catalog 00-4506-51) and monensin (BD Biosciences, catalog 554724) were added after 2 hours. After 6 hours of culturing, cells were washed and stained with the LIVE/DEAD Fixable Aqua Dead Cell Stain Kit (Thermo Fisher Scientific, catalog L34957) in PBS for 30 minutes at room temperature. FcR blocking was conducted for 15 minutes at room temperature with 10% normal mouse IgG (Thermo Fisher Scientific, catalog OB2040-09) in staining buffer (PBS containing 0.1% NaN*3* and 1.7% BSA). Cell-surface staining was conducted using cocktails of the following fluorescently labeled BD Biosciences antibodies: CD20 PercP-Cy5.5 (catalog 560736); CD3 AF700 (catalog 557943); CD14 BV421 (catalog 563743); HLA-DR BV480 (catalog 566113); CD69 BV605 (catalog 562989); CD16 BUV496 (catalog 612944); and CD56 PE-Cy7 (catalog 557747) in Brilliant Stain Buffer (Thermo Fisher Scientific, catalog 00-4409-42) for 30 minutes at room temperature. Intracellular staining was performed after fixation and permeabilization using the FIX & PERM Cell Permeabilization Kit (Thermo Fisher Scientific, catalog GAS004)) and a cocktail of fluorescent antibodies against the intracellular proteins IFN-γ BV711 (BD Biosciences, catalog 564039) and TNF-α BV650 (BD Biosciences, catalog 56930). Flow cytometric data were collected on a FACSymphony A5 flow cytometer (BD) and analyzed using FlowJo, version 10.8.1 software for Mac OS (BD).

### Translocated microbial products composition.

Circulating total RNA and DNA were isolated from frozen plasma of 25 HESC and 24 HESN participants using the RNAzol BD Column Kit (Molecular Research Center [MRC]) according to the manufacturer’s protocol. Shotgun sequencing was performed as previously described (38). Briefly, isolated plasma RNA was converted to cDNA using random hexamers, and then both the cDNA and the isolated plasma DNA were used to individually generate Illumina-ready libraries with the NEBNext Ultra II RNA Library Prep Kit for Illumina (New England Biolabs). The final libraries were validated using a TapeStation 4200 instrument (Agilent Technologies) and then sequenced on a NovaSeq 6000 (Illumina) using 151 bp paired-end reads. To control for contamination, 2 water samples were processed along with plasma samples from extraction of nucleic acids through sequencing.

Demultiplexed samples were analyzed using the CZID (https://czid.org/) server (49). DNA and RNA fractions were prepared and sequenced separately. Analysis was performed independently on the 2 datasets. A background model for each dataset was generated using 2 no-template control (NTC) samples for each DNA and RNA subsets. Sample taxonomic reports were generated using the background model that assigns a *z* score to a pathogen call. The range of scores was –100 to 100. All pathogen calls with a *z* score below 0 were filtered out.

To test the difference between HESN and HESC individuals at the kingdom and phylum taxonomic levels, 2-sided *t* tests were carried out using the reads per million (RPM) counts. CZID exported the counts data at the species level, which were summed per individual within the 2 taxonomic levels, and the summed counts were used for hypothesis testing. Counts for only those species that had an above-zero CZID *z* score were considered. The Vegan package in R, version 4.4.1, was used to calculate Shannon diversity, evenness, and richness based on filtered RPM counts for DNA and RNA from the CZID output. PCA was carried out in R using filtered RPM counts for DNA and RNA per organism call from the CZID output across all study participants, grouping the organisms into their respective kingdoms. The PCA dot plots were generated using the first 2 PCs for each individual and colored according to HESN and HESC groupings. The first 15 PCs from each PCA were used to conduct a Spearman’s rank correlation with the cell population frequencies from the flow cytometric analysis. To test the difference between HESN and HESC at the kingdom and phylum taxonomic levels, 2-sided *t* tests were carried out using the RPM counts. Tests were performed on 2 sets of data, first with only the *z* score–filtered data and the second with a subset of genera that are part of the gut microbiome as listed by Leviatan et al. (39). Spearman’s rank correlations were performed for the 2 datasets between the RPM count data and the cell population frequencies from the flow cytometric analysis. Heatmaps were drawn to display the cell populations on the columns and phylum on the rows. Cells were colored according to rho values and marked with an asterisk(s) to show if the correlation test was significant at a *P* value of less than 0.05.

### Statistics.

Statistical analysis was performed using GraphPad Prism, version 9.4.1 for Mac OS (GraphPad Software). Comparisons between groups were performed using the Mann-Whitney *U* test unless otherwise indicated. Spearman’s test was used to calculate correlations between cell phenotypes and peak or set-point viral load. *P* values below 0.05 were considered significant. For violin plots, by definition, width of the shape is proportional to the distribution of the data on the *y* axis.

Logistic regression analysis was performed with the dependent variable set as HIV status (HESC/HESN), while the independent variables included the 8 phenotypical markers found to be statistically significant in the univariate analysis. Bidirectional stepwise regression, which combines forward selection and backward elimination, was used. The selection was based on Akaike information criterion (AIC). Starting with the full model, variables were iteratively added or removed until no further improvement in AIC was observed. ORs were computed for each marker to estimate their effect on the likelihood of HIV acquisition, with 95% CIs providing precision of these estimates. A random forest model was applied to assess the importance of each variable, evaluating the variance explained by each factor and ranking them on the basis of the mean decrease Gini coefficient. Receiver operating characteristic (ROC) curves were constructed to evaluate the logistic regression model’s predictive performance, with the AUC measuring the model’s ability to discriminate between HESC and HESN. An AUC value closer to 1 indicated better model performance. Logistic regression, random forest, and ROC curve analyses were conducted using R, version 4.3.2, and the relevant packages.

### Study approval.

The investigators have adhered to the policies for protection of human participants as prescribed in applicable DOD, NIH and Thai policies and regulations. The RV217 Study was approved by the Walter Reed Army Institute of Research in the United States and the IRBs of the Kenya Medical Research Institute Ethics Review Committee (Nairobi, Kenya), National Institute for Medical Research (Dar es Salaam, Tanzania), Mbeya Medical Research Ethics Committee (Mbeya, Tanzania), Uganda National Council for Science and Technology National HIV/AIDS Research Committee (Kampala, Uganda), and Royal Thai Army Medical Department (Bangkok, Thailand). All study participants gave written informed consent.

### Data availability.

All supporting data are available in the Supporting Data Values file.

## Author contributions

DPP, BMS, DCD, and SJK conceptualized the study. KM, KFN, LM, NSL, IS, SKM, JRC, DPP, ARH, and FL performed experiments. KM, KFN, RF, SG, and DK conducted analyses. KM, KFN, and DPP wrote the manuscript. LAE and MLR, and members of the RV217 cohort study team were responsible for administration of the study. DPP, SV, JAA, SJK, MLR, and DCD supervised the study. All authors have reviewed, edited, and approved the manuscript.

## Supplementary Material

Supplemental data

Supporting data values

## Figures and Tables

**Table 1:**
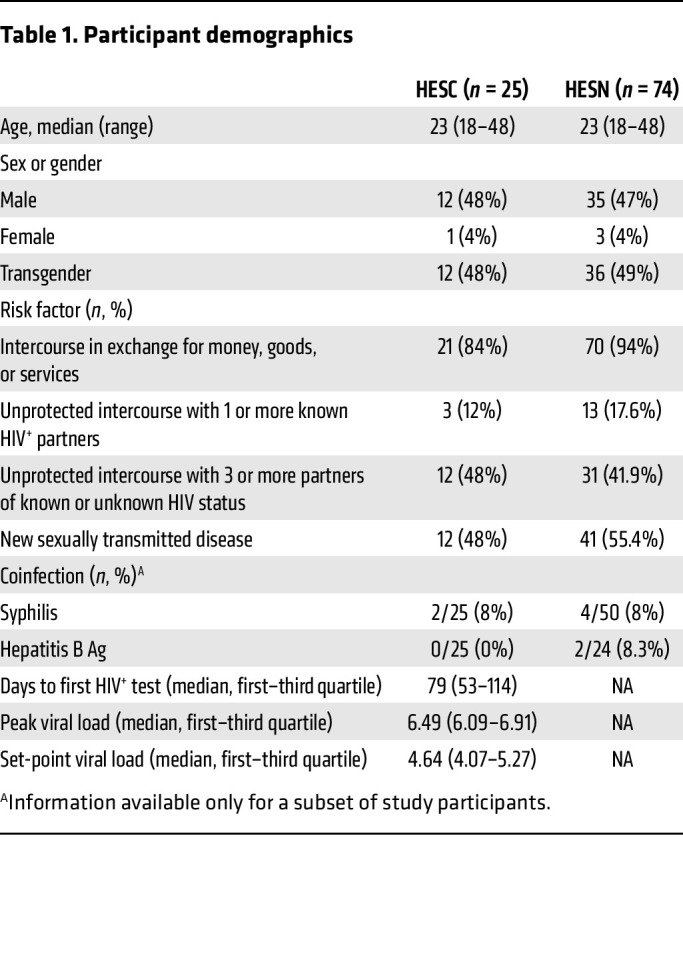
Participant demographics

## Version 1 05/08/2025

In-Press Preview

## Version 2 08/01/2025

Electronic publication
