# Supplementary material for: NK cell activation and CD4+ T cell α4β7 expression are associated with susceptibility to HIV-1
Source: J Clin Invest. 2025 May 8;135(15):e187992. doi: 10.1172/JCI187992 (PMC12321385; doi:10.1172/JCI187992)
Supplement: Supplemental data [file jci-135-187992-s134.pdf]

The RV217 study group includes the following team members from **U.S. Military HIV Research Program, Walter Reed Army Institute of Research, Silver Spring, Maryland 20910, USA**: Shelly J. Krebs, Morgane Rolland, Sodsai Tovanabutra, Eric Sanders-Buell, Vicky R. Polonis, Bonnie Slike, Matthew Johnston, Susan Mason, Mark Milazzo, Qun Li, Mangala Rao, Rasmi Thomas; from **Diagnostics and Countermeasures Branch Laboratory Director, Walter Reed Army Institute of Research, Silver Spring, Maryland 20910, USA**: Sheila Peel, Linda Jagodozinski, Jennifer Malia; from **Armed Forces Research Institute of Medical Sciences, Bangkok, Thailand**: Rapee Trichavaroj, Alexandra Schuetz, Siriwat Akapirat, Somchai Sriplienchan; from **Makere University Walter Reed Project, Kampala, Uganda:** Hannah Kibuuka, Prossy Sekiziyivu , Monica Millard; from **Kenya Medical Research Institute/U.S. Army Medical Research Directorate-Africa, Kericho, Kenya**: Fred Sawe, Ibrahim Daud, Josphat Kosgei; from **National Institute for Medical Research-Mbeya Medical Research Center, Mbeya, Tanzania**: Lucas Maganda, Nyanda Ntinginya, Cornelia Lueer, Abisai Kisinda; from **German Center for Infection Research (DZIF), partner site Munich, Germany and Division of Infectious Diseases and Tropical Medicine, Medical Center of the University of Munich (LMU), Munich, Germany**: Inge Kroidl, Michael Hoelscher, Arne Kroidl, Christoff Geldmacher; from **Division of AIDS, NIAID, NIH, Bethesda, Maryland 20852, USA**: Mary Marovich, Edith Swann, Michael A. Eller; from **EMMES Corporation, Rockville, Maryland 20850, USA**: Peter Dawson.

Supplementary Table 1: list of markers used for flow cytometry

Marker	Clone	Fluorochrome	Manufacturer	Panel
FcRg	NP_004097	FITC	Millipore	NK
CD158a/h/g (KIR2DL1/S1/S3/S5)	HP-MA4	BB700	BioLegend	NK
CD158e1 (KIR3DL1)	DX9	BB700	BioLegend	NK
NKG2D	1D11	BB790-P	BD	NK
a4b7	Act-1	AF647	In-house	NK
NKp30	p30-15	R718	BD	NK
CD328 (Siglec 7)	REA214	APC-Vio770	Miltenyi	NK
KLRG1	SA231A2	BV421	BioLegend	NK
HLA-DR	G46-6	BV480	BD	NK
Live/Dead		Fixable AQUA	ThermoFisher	NK
NKp80	5D12	BV650	BD	NK
NKp46	9E2/NKp46	BV711	BD	NK
Ki67	B56	BV750	BD	NK
CD33	WM53	BV786	BD	NK
CD19	SJ25C1	BV786	BD	NK
CD3	UCHT1	BV786	BD	NK
CD57	NK-1	BUV395	BD	NK
CD16	3G8	BUV496	BD	NK
CD56	B159	BUV563	BD	NK
ILT2	GHI/75	BUV615	BD	NK
PD-1	EH12.1	BUV661	BD	NK
CD38	HB7	BUV737	BD	NK
CD94	HP-3D9	BUV805	BD	NK
EOMES	WD1928	PE	ThermoFisher	NK
NKG2c	REA205	PE-Vio615	Miltenyi	NK
T-Bet	4B10	PE Cy 5	BD	NK
NKG2A (CD159a)	REA110	PE-Vio770	Miltenyi	NK
Va24	C15	BB515	Beckman Coulter	Innate T cell
CCR6	11Ag	BB630-P	BD	Innate T cell
CD69	FW50	BB660-P2	BD	Innate T cell
CXCR5	R58B2	BB790	BD	Innate T cell
a4b7	Act-1	AF647	In-house	Innate T cell
CD161	DX12	R718	BD	Innate T cell
CD8	RPA-T8	APC Cy 7	BD	Innate T cell

MR1 Tetramer		BV421	NIH tetramer facility	Innate T cell
HLA-DR	G46-6	BV480	BD	Innate T cell
Live/Dead		Fixable AQUA	ThermoFisher	Innate T cell
CD19	SJ25C1	BV570	BD	Innate T cell
CD14	M5E2	BV570	BD	Innate T cell
Granzyme B	GB11	BV605	BD	Innate T cell
CCR7	2-L1-A	BV650	BD	Innate T cell
X-pan TCRgd	11F2	BV711	BD	Innate T cell
Ki67	B56	BV750	BD	Innate T cell
CD45RO	UCHL1	BV786	BD	Innate T cell
CD57	NK-1	BUV395	BD	Innate T cell
CD16	3G8	BUV496	BD	Innate T cell
X-Vd2 TCR	B6	BUV563	BD	Innate T cell
CD56	B159	BUV615-P	BD	Innate T cell
PD-1	EH12.1	BUV661	BD	Innate T cell
CD38	HB7	BUV737	BD	Innate T cell
CD4	SK3	BUV805	BD	Innate T cell
Vb11	C21	PE	Beckman	Innate T cell
Va7.2	3C10	PE-Dazzle594	BioLegend	Innate T cell
T-bet	4B10	PE Cy 5	BD	Innate T cell
CD3	SK7	PE Cy 5.5	ThermoFisher	Innate T cell
TCRd1	TS8.2	PE Cy 7	ThermoFisher	Innate T cell

Supplementary Figures

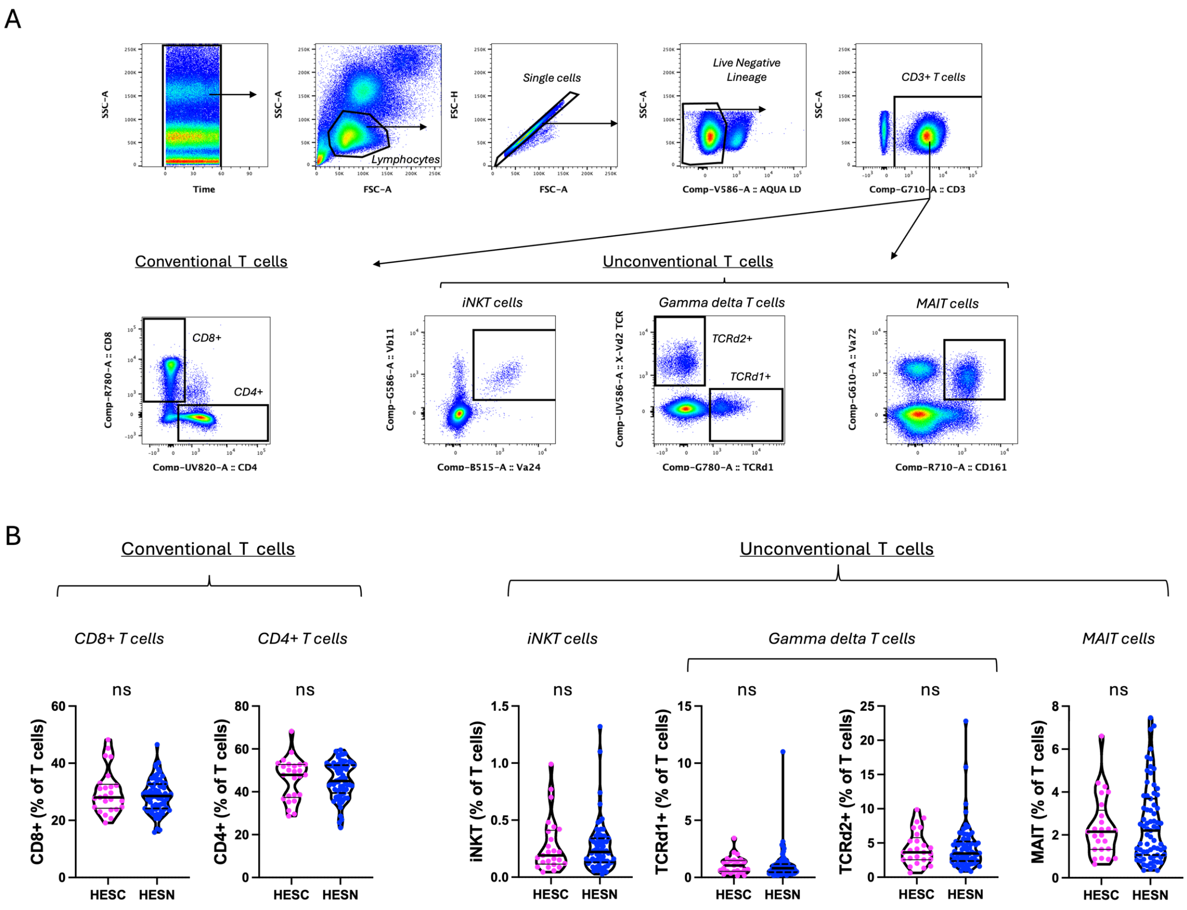

Supplementary Figure 1. Gating strategy for conventional and unconventional T cells (A). Frequencies of conventional CD4 and CD8 T cells, iNKT cells, $\gamma\delta$ T cells, and MAIT cells in HESC prior to seroconversion (N=25) and HESN (N=74) (B). Mann–Whitney test.

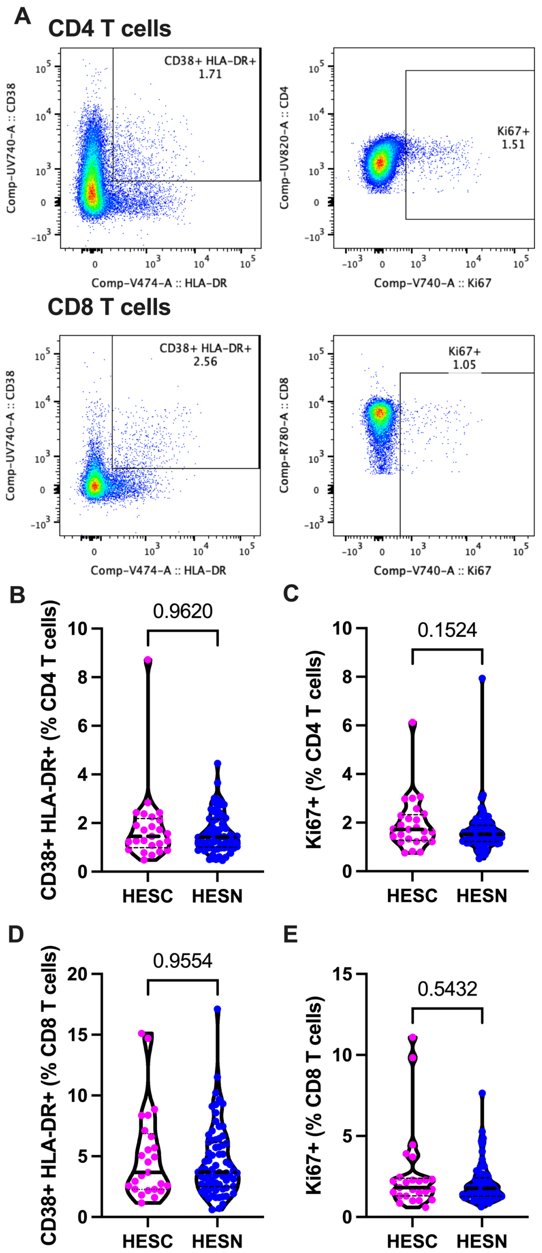

Supplementary Figure 2. Representative flow plots showing CD38 and HLA-DR co-expression (left) as well as Ki67 (right) by CD4 (top) and CD8 (bottom) T cells. Violin plots showing the levels of co-expression of CD38 and HLA-DR (A, C) and Ki67 (B, D) by conventional CD4 (A, B) and CD8 (C, D) T cells in HESN and HESC prior to HIV-1 acquisition. Mann–Whitney test.

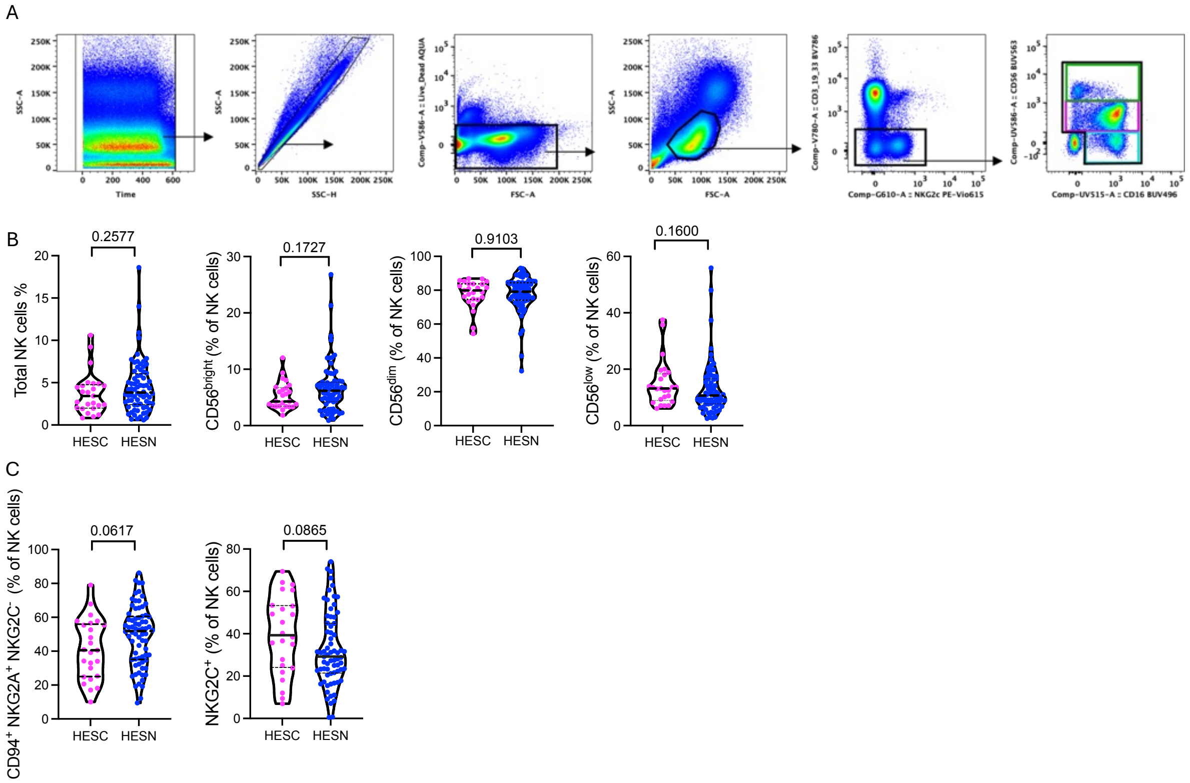

Supplementary Figure 3. Gating strategy for NK cells (A). Frequency of total NK cells, CD56^{bright}, CD56^{dim}, and CD56^{low} NK cells in HESC prior to HIV-1 acquisition (N=25) and HESN (N=74) (B). Mann–Whitney test.

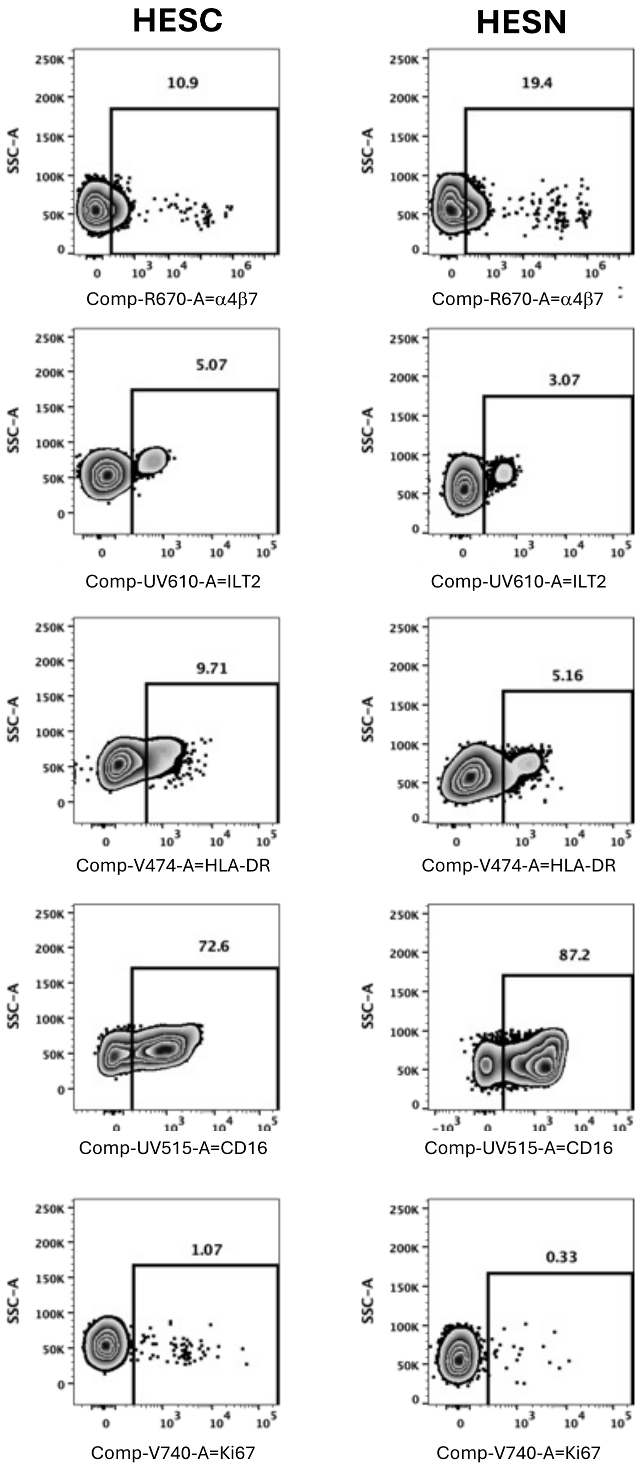

Supplementary Figure 4. Representative flow plots from one HESC and HESN showing expression of $\alpha 4\beta 7$, ILT2, HLA-DR, CD16, and Ki67 by NK cells.

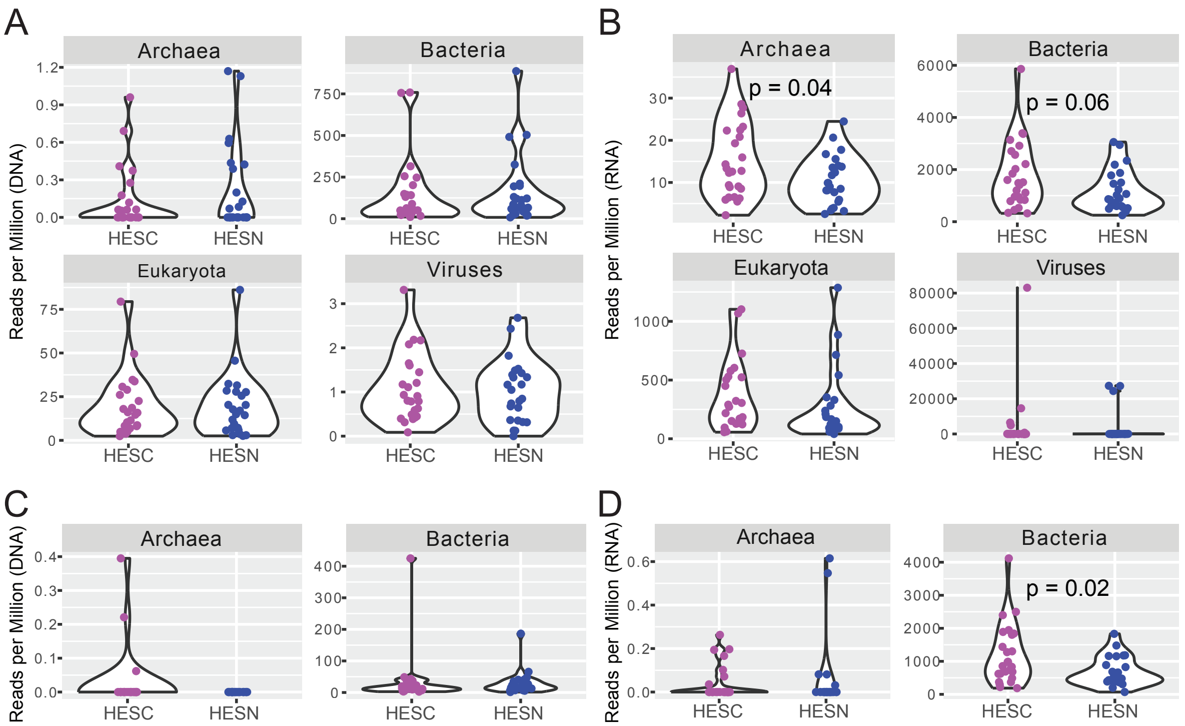

Supplementary Figure 5. Violin plots showing the relative abundance as reads per million of the microbial products in plasma at the kingdom level for DNA (A) and RNA (B). Plots showing the relative abundance of the gut-associated microbial products in plasma at the kingdom level for DNA (C) and RNA (D). HESC N=25 prior to HIV-1 acquisition and HESN N=24. T-test.

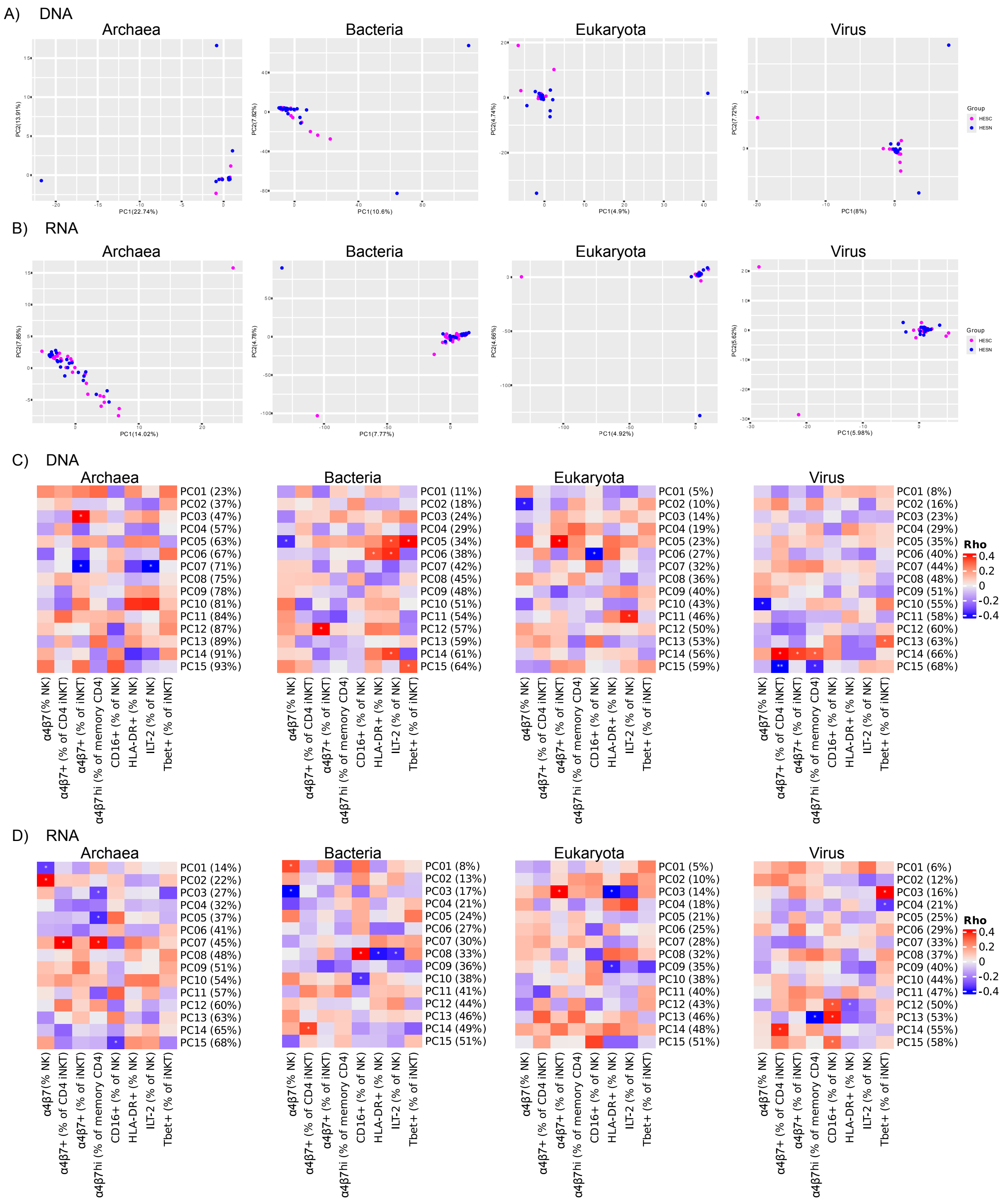

Supplementary Figure 6. PCA plots of the composition of microbial products in plasma at the kingdom level for DNA (A) and RNA (B). Heat map showing the Spearman rho values for association between the PCA of translocated microbial products for each kingdom and immune phenotype for DNA (C) and RNA (D). The cumulative variance explained by each PCA is indicated in bracket. HESC N=25 prior to HIV-1 acquisition and HESN N=24. P values below 0.05 are indicated by a *.
